# A causal relationship between leukocyte telomere length and multiple sclerosis: A Mendelian randomization study

**DOI:** 10.3389/fimmu.2022.922922

**Published:** 2022-07-15

**Authors:** Qiao Liao, Jian He, Fa-Fa Tian, Fang-Fang Bi, Kun Huang

**Affiliations:** ^1^ Department of Neurology, Xiangya Hospital, Central South University, Changsha, China; ^2^ National Clinical Research Center for Geriatric Disorders, Xiangya Hospital, Central South University, Changsha, China; ^3^ Department of Gastroenterology, Nanfang Hospital, Southern Medical University, Guangzhou, China; ^4^ Institute of Molecular Precision Medicine and Hunan Key Laboratory of Molecular Precision Medicine, Xiangya Hospital, Central South University, Changsha, China

**Keywords:** telomere, telomere length, multiple sclerosis, Mendelian randomization study, neuroimmunology

## Abstract

**Objectives:**

Multiple sclerosis (MS) is a chronic inflammatory autoimmune and degenerative disorder of the central nervous system. Telomeres are protective structures located at the ends of linear chromosomes, and leukocyte telomere length (LTL) is closely connected with cell aging and senescence. However, the relationship between LTL and the risk of MS remains unknown.

**Methods:**

We performed a two-sample Mendelian randomization (MR) to evaluate whether LTL was causally associated with MS risk.

**Results:**

In our MR analysis, 12 LTL-related variants were selected as valid instrumental variables, and a causal relationship between LTL and MS was suggested. The risk of MS nearly doubled as the genetically predicted LTL shortened by one standard deviation (SD) under the inverse variance weighted (IVW) fixed effect model (odds ratio (OR) = 2.00, 95% confidence interval (CI): 1.52-2.62, *p* = 6.01e-07). Similar estimated causal effects were also observed under different MR models. The MR–Egger regression test did not reveal any evidence of directional pleiotropy (intercept = -0.005, stand error (SE) = 0.03, *p* = 0.87). The Mendelian Randomization Pleiotropy RESidual Sum and Outlier (MR-PRESSO) analysis also indicated no directional pleiotropy or outliers for any LTL-related IVs (*p*-global test = 0.13). In addition, a leave-one-out sensitivity analysis showed similar findings, which further emphasized the validity and stability of the causal relationship.

**Conclusions:**

Our results suggest a potential causal effect of LTL on the risk of MS. Genetically predicted shorter LTL could increase the risk of MS in the European population. LTL should be noted and emphasized in the pathogenesis and treatment of MS.

## Introduction

Multiple sclerosis (MS) is a chronic inflammatory autoimmune and degenerative disorder of the central nervous system (1). The exact cause of MS is still unknown, although it is widely accepted that genetic susceptibility and environmental factors play essential roles in the onset and pathogenesis of MS. Despite MS has a predominant prevalence among young adults, the number of older patients with MS is also growing (2). Aging has exerted a profound effect on the pathological changes and clinical course of MS (3). Immunosenescence, which is defined as natural age-associated immunity and closely related to the aging of inflammatory function, may contribute to the higher possibility of developing such pathological alterations. In addition, immunosenescence has been related to chronological age (4). Understanding the role of immunosenescence in MS onset is of paramount significance to uncover the very nature of MS pathogenesis.

Telomeres are protective structures located at the ends of linear chromosome and consist of two core components, including repeated DNA sequences and specific DNA binding proteins (5). The length of telomeres gradually decreases with cell divisions and aging and finally reaches a critical length, after which the cell becomes senescent (6). Therefore, telomere length (TL) has become the most widely recognized measurement of an individual’s biological age.

Telomere shortening is related to dysregulated immune function and a hallmark of immunosenescence (4). Recently, a growing number of studies concerning the role of LTL in MS have come into view (7, 8). A cross-sectional study revealed that the LTL decreases over time in both relapsing-remitting and primary progressive MS patients, and patients with shorter TL at baseline harbor a higher chance of converting from relapsing-remitting MS (RRMS) to secondary progressive multiple sclerosis (SPMS) (9). Meanwhile, an observational study demonstrated that shorter LTL is significantly correlated, independent of age, with higher scores of the Expanded Disability Status Scale (EDSS) and lower brain volume, implying the role of LTL in the disability progression of MS (10, 11). Increasing evidence regarding LTL alterations in MS has emphasized that biological aging may play a role in neurological injury in MS. Further exploration of the role of LTL in MS will promote a deeper understanding of the pathogenesis of the disease and provide more therapeutic targets for MS. We presumed that a shorter LTL could increase the risk of MS. Thus, preventing the loss of LTL could be a novel approach to reduce the risk of MS.

Two-sample Mendelian randomization (MR) analysis is widely adopted to draw causal inferences of the effect of an exposure on an outcome (12). Therefore, in the current study, we performed a two-sample MR to evaluate whether LTL is causally associated with MS risk by using genetic variants that are strongly related to LTL as instrumental variables and summary statistics from large genome-wide association study (GWAS) databases.

## Methods

### Data sources

We performed a standard two-sample MR to investigate the causal relationship between LTL and MS. Genetic variants associated with LTL were used as instrumental variables (IVs), and the interactions of IVs-LTL and IVs-MS were derived from different and nonoverlapping datasets from available genome-wide association studies (GWASs). In our MR analysis, we used the public available summary-data of LTL and MS from open GWAS database, and the ethics vote is not applicable in our study.

For the LTL, summary GWAS results were obtained from a genome-wide meta-analysis that enrolled a total of 78,592 European-descent participants (13). It was the most updated and largest LTL GWAS meta-analysis, which included three different study cohorts as follows: European Prospective Investigation into Cancer and Nutrition (EPIC) InterAct (n=19,779), EPIC Cardiovascular Disease (CVD) (n=11,915), and European Network for Genetic and Genomic Epidemiology (ENGAGE) consortium (n=46,898). The EPIC-InterAct study aimed to investigate the effects of genetic and behavioral risk factors on type 2 diabetes risk (14, 15). EPIC-CVD was designed as a case-cohort study with a focus on incident coronary heart disease and stroke events (16). ENGAGE consortium contains cohorts focusing on different diseases including coronary artery disease, metabolic syndrome, psychiatric (depression/anxiety) and general population (13, 17). Age-, sex- and cohort-specific covariates were adjusted in the LTL GWAS meta-analysis. Mean LTL measurements were conducted using an established quantitative PCR technique and expressed as a ratio of the telomere repeat number (T) to a single-copy gene (S). LTL measurements were standardized by using a calibrator sample or by quantifying against a standard curve. In total, 20 significant SNPs were identified to be associated with LTL at a level of genome-wide significance (*p* < 5.00e-8).

MS GWAS statistics, including 47,429 MS patients and 68,374 controls, were obtained from the publicly available GWAS summary datasets developed by MRC Integrative Epidemiology Unit (IEU) at the University of Bristol (18–20). The authors have performed a meta-analysis of 15 genome-wide MS datasets and replicated the genetic associations in independent large population.

### Genetic instruments for telomere length

**Figure 1:**
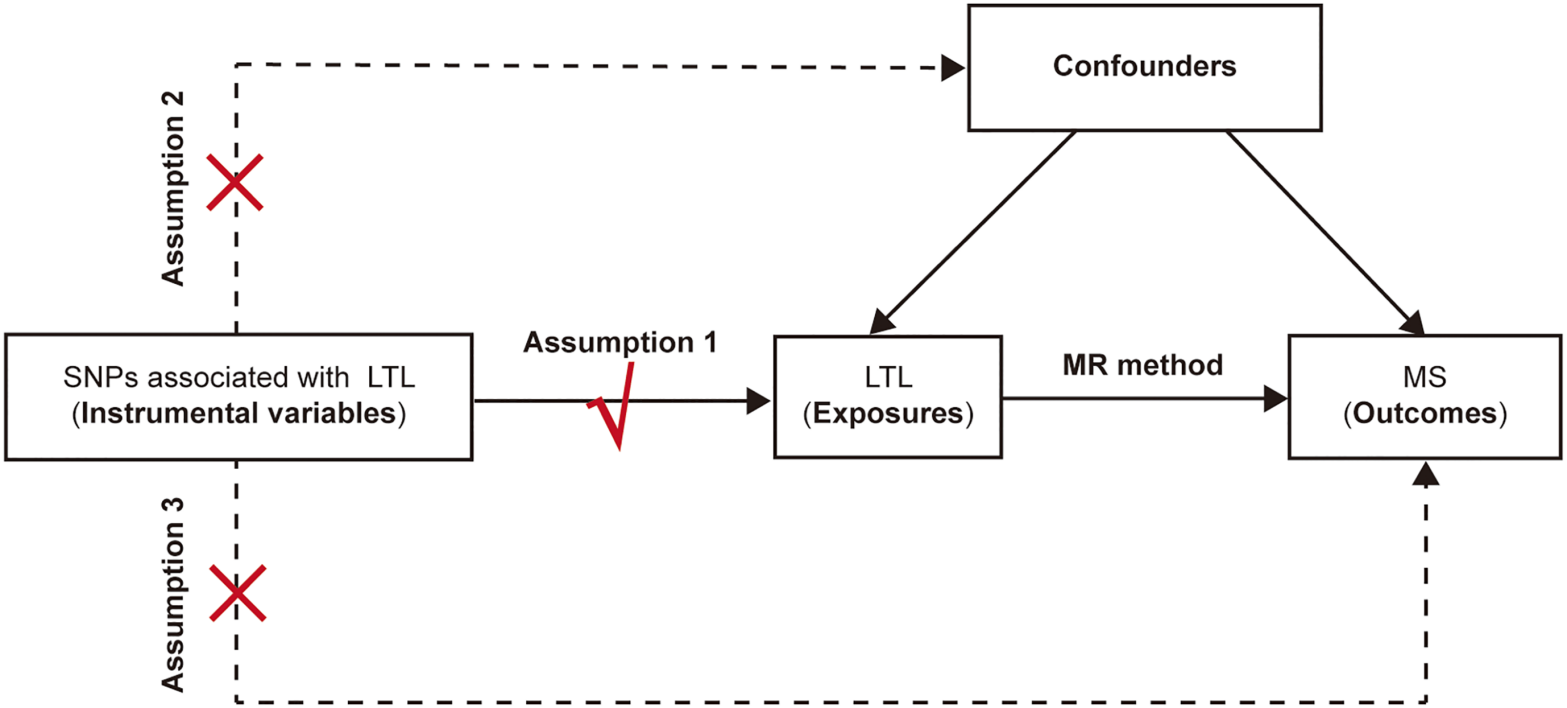
The schematic diagram for the Mendelian randomization analysis. SNP, single-nucleotide polymorphism; LTL, leukocyte telomere length; MR, Mendelian randomization; MS, multiple sclerosis.

Three fundamental conditions of MR analysis should be satisfied to ensure the validity of genetic variants as IVs (**Figure 1**), listed as follows: (1) the selected IVs are significantly associated with the exposure (LTL); (2) the available IVs are not associated with any confounders of the exposure (LTL)-outcome (MS) association; and (3) the selected IVs do not affect the outcome (namely, MS), except possibly *via* the association with the exposure (namely, the LTL). Previous studies on MS have demonstrated that vitamin D insufficiency (21, 22), smoking (23) and higher body mass index (BMI) (24) are associated with increased risk of MS. Moreover, it turns out that vitamin D (25), smoking (26) and BMI (27) are also associated with telomere length. Therefore, to make sure the selected SNPs are in accordance with the second core assumption of two-sample MR, we excluded SNPs that are associated with vitamin D traits, smoke status and BMI traits. Besides, we also omitted the SNPs associated with MS based on the third assumption. In general, the SNPs selected as instrumental variables in our analysis are consequently guaranteed to reduce the bias produced by confounding factors.

For the LTL GWAS results, 20 SNPs were robustly associated with LTL at a genome-wide level (*p* < 5.00e-8). After linkage disequilibrium analysis with a cutoff for *r* ^2^ of 0.001, a base pair window of 10,000 kb, and the removal of SNPs associated with confounders and outcomes based on the PhenoScanner V2 database (http://www.phenoscanner.medschl.cam.ac.uk/), only 15 SNPs remained that fulfilled the three core consumptions and were selected as IVs to assess the causal relationship between LTL and MS. SNPs that were not found in the MS GWAS were replaced by their proxies, which were identified from LDlink (https://ldlink.nci.nih.gov/). The directions of SNP effects on LTL and MS were harmonized, and palindromic SNPs were omitted in the MR analysis.

### Statistical analysis

The inverse variance weighted (IVW) method was motivated as a meta-analysis of the variant-specific causal estimates and was adopted to obtain a pooled estimation of the causal effect. The IVW method has been widely used in Mendelian randomization analysis, especially when the enrolled IV satisfies the assumptions that all IVs in the analysis are of robust validity. In addition, the MR–Egger method was utilized to provide a less biased causal estimation when weak IVs existed. In addition, the median-based method was applied to eliminate the influence of abnormal SNPs on the causal estimation, while the maximum likelihood method contributed to assessing the overlap in the population. For individual SNPs, estimated causal effects were expressed as the odds ratios (ORs) from the Wald ratio method.

### Sensitivity analysis

After MR analysis, sensitivity analysis, including heterogeneity and pleiotropy, was performed. Heterogeneity between the SNP-specific causal estimates can be assessed using approaches from meta-analyses. Therefore, Cochran’s *Q* statistic was calculated to measure heterogeneity among the estimated effects of individual LTL-associated SNPs on MS. A large Q statistic value indicates that the effect of individual LTL-associated SNPs on MS differs, which suggests heterogeneity and that the findings should be interpreted with caution. To explore whether the LTL-associated SNPs are pleiotropic, the MR–Egger regression test was adopted to identify possible pleiotropy, and a *p* value of the MR–Egger intercept greater than 0.05 suggested a lack of horizontal pleiotropy. In addition, MR-PRESSO (Mendelian Randomization Pleiotropy RESidual Sum and Outlier) analysis was performed to detect widespread horizontal pleiotropy in causal relationships. Furthermore, a radial plot was drawn to vividly illustrate the causal estimate. In addition, leave-one out analysis was used to recognize the possible LTL-associated SNPs that might influence the causal effect and identify potential outliers.

Our two-sample MR analysis was conducted using R software (version 4.1.1). The packages ‘TwoSampleMR’ (https://github.com/MRCIEU/TwoSampleMR) and ‘forestplot’ (https://cran.r-project.org/web/packages/forestplot/index.html) were used for statistical analysis, data output and visualization. All presented *p* valuess were two tailed, and *p* values less than 0.05 were considered statistically significant.

## Results

In our two-sample MR analysis, 15 LTL-related SNPs in conformity with the three core assumptions were primarily chosen as IVs after the clump process and the removal of SNPs associated with confounding factors and MS according to the PhenoScanner V2 database. Three LTL-related SNPs that were not identified in the MS GWAS were replaced by their proxies. After harmonization of the alleles and effects between LTL and MS and the removal of SNPs for being palindromic with intermediate allele frequencies, only 12 LTL-related SNPs were considered as IVs for LTL-MS associations and ultimately remained in the MR analysis (**Table 1**).

**Table 1:** Characteristics of 15 LTL-related SNPs selected as IVs for LTL-MS associations.

SNP	Chr	Position	EA	OA	EAF	β	SE	*p* value
rs3219104	1	226562621	C	A	0.83	0.042	0.006	9.60e-11
rs55749605	3	101242614	A	C	0.58	-0.037	0.007	2.45e-08
rs10936600*	3	169514585	T	A	0.24	-0.086	0.006	7.18e-51
rs13137667	4	71774347	C	T	0.96	0.077	0.014	2.43e-08
rs4691895*	4	164048199	C	G	0.78	0.058	0.006	1.58e-21
rs7705526	5	1285974	A	C	0.33	0.082	0.006	5.34e-45
rs59294613	7	124654217	A	C	0.29	-0.041	0.006	1.17e-13
rs9419958	10	105675946	C	T	0.86	-0.064	0.007	5.05e-19
rs228595	11	108105593	A	G	0.42	-0.029	0.005	1.43e-08
rs2302588*	14	73404752	C	G	0.1	0.048	0.008	1.68e-08
rs3785074	16	69406986	G	A	0.26	0.035	0.006	4.64e-10
rs62053580	16	74680074	G	A	0.17	-0.039	0.007	4.08e-08
rs7194734	16	82199980	T	C	0.78	-0.037	0.006	6.94e-10
rs8105767	19	22215441	G	A	0.3	0.039	0.005	5.42e-13
rs75691080	20	62272248	T	C	0.09	-0.067	0.009	5.99e-14

*, Palindromic SNPs that are not kept in the MR analysis.

LTL, leukocyte telomere length; SNP, single-nucleotide polymorphism; IV, instrumental variable; MS, multiple sclerosis; EA, effect allele; OA, other allele; EAF, effect allele frequency; β, the per-allele effect on LTL measurement; SE, standard error.

### Genetically predicted shorter LTL is associated with increased risk of MS

**Figure 2:**
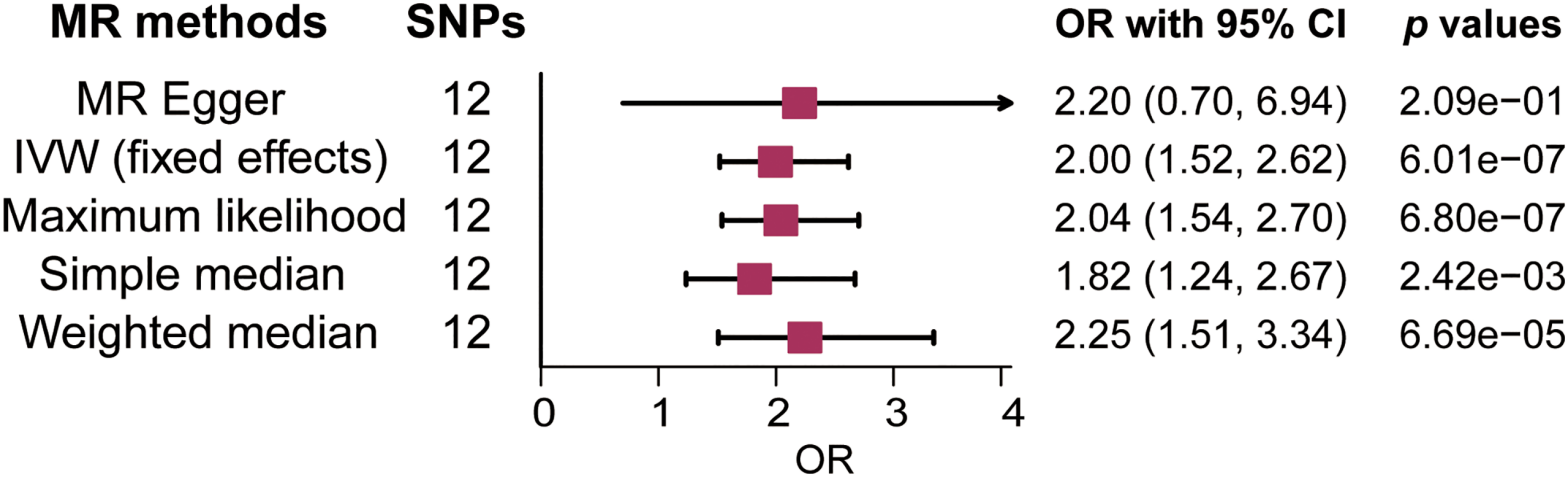
Forest plot of the association between genetically predicted leukocyte telomere length (LTL) and multiple sclerosis (MS). OR means a change in MS risk associated with a 1- standard deviation decrease in genetically determined LTL. SNP, single-nucleotide polymorphism; OR, odds ratio; CI, confidence interval; IVW, inverse variance weighted.

**Figure 3:**
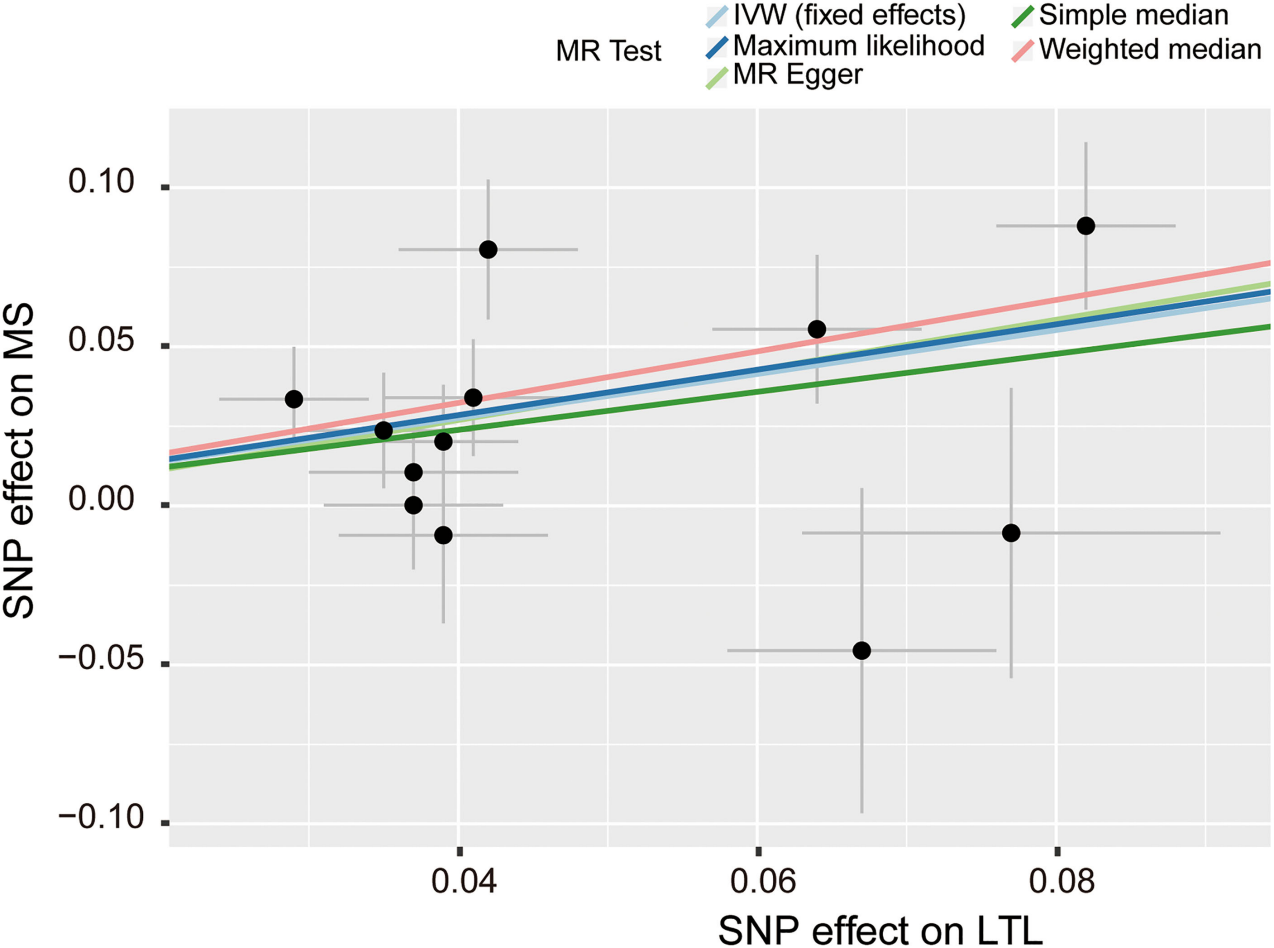
Scatter plot of the effect size and 95% CI of each SNP on LTL and MS risk. The horizontal axis reflects genetic effect of each SNP on LTL. The vertical axis represents the genetic effect of each SNP on MS risk. SNP, single-nucleotide polymorphism; MS, multiple sclerosis; LTL, leukocyte telomere length; IVW, inverse variance weighted.

There was no clear evidence of heterogeneity between individual LTL-related SNPs, as the MR Egger intercept test harbored a Cochran’s Q statistic of 17.189 with a *p* value larger than 0.05 (*p*=7.00e-02). Therefore, the IVW method with a fixed-effect model was adopted for causal estimations. As shown in **Figure 2**, the risk of MS nearly doubled as the genetically predicted LTL shortened by one standard deviation (SD) under the IVW fixed effect model (OR=2.00, 95% CI: 1.52-2.62, *p*=6.01e-07). In addition, similar estimated causal effects were observed under different MR methods, including the maximum likelihood (OR=2.04, 95% CI: 1.54-2.70, *p*=6.80e-07), simple median (OR=1.82, 95% CI: 1.24-2.67, *p*=2.42e-03), and weighted median method (OR= 2.25, 95% CI: 1.51-3.34, *p*=6.69e-05). The causal link estimation based on the MR–Egger method demonstrated a similar trend of the effect of LTL on MS, although there was no statistical significance (OR=2.20, 95% CI: 0.70-6.94, *p*=2.09e-01). Therefore, the MR analysis suggested that genetically determined shorter LTL was associated with an increased risk of MS (**Figure 3**).

### Pleiotropy and sensitivity analysis

**Figure 4:**
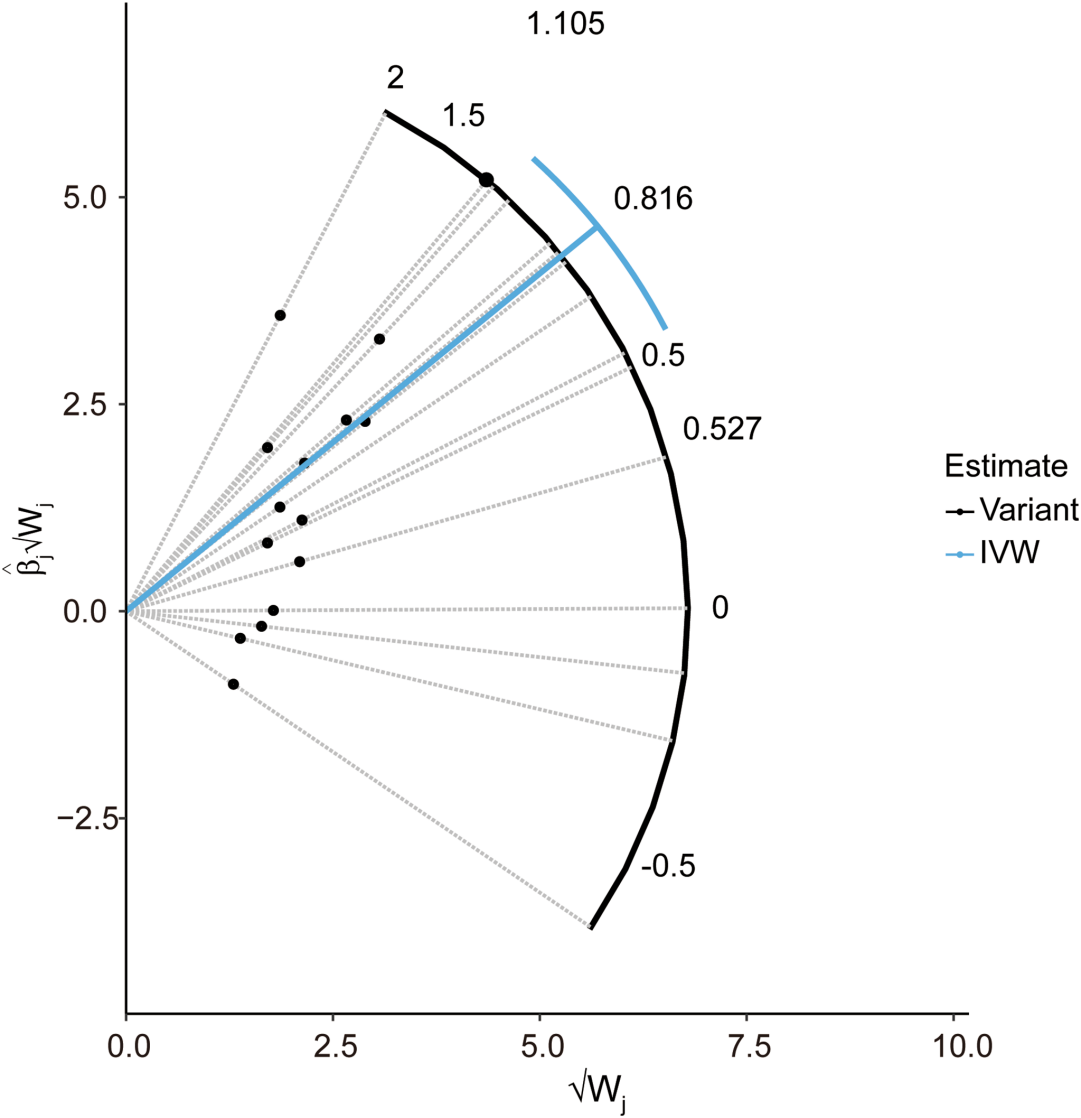
Radial plot to illustrate each individual outlier SNPs for the effect of LTL on MS risk. The radial curve demonstrates the estimated ratio for each individual SNP, as well as the overall IVW estimate (in blue). Black dots indicate valid SNPs. IVW, inverse variance weighted method; SNP, single-nucleotide polymorphism; MR, Mendelian randomization; MS, multiple sclerosis; LTL, leukocyte telomere length.

**Figure 5:**
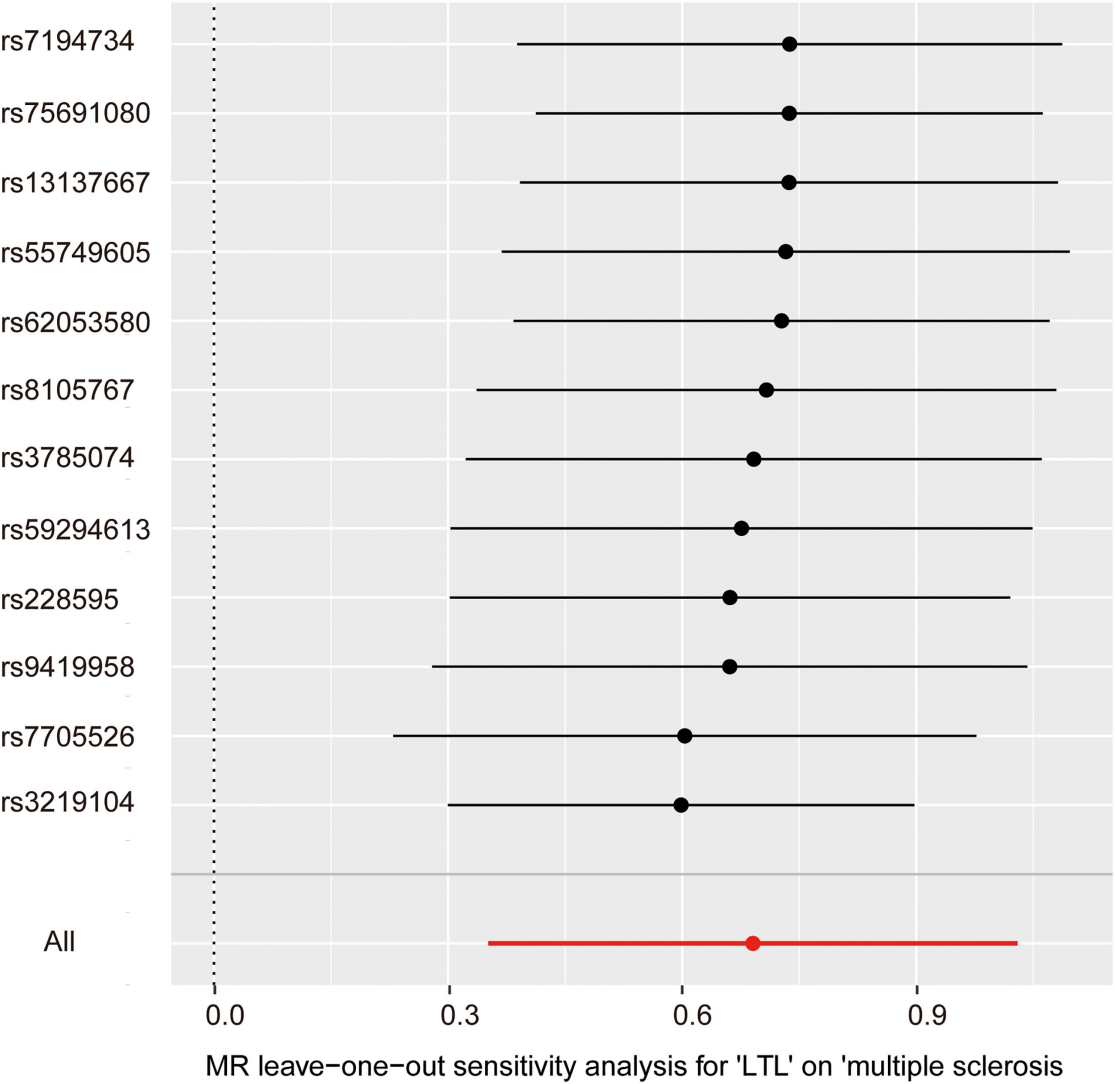
Leave-one-out analysis for the impact of individual SNPs on the association between LTL and MS risk. By leaving out exactly one SNP, it shows how each individual SNP influences the overall estimate.

In the pleiotropy analysis, the MR–Egger regression test did not reveal any evidence of directional pleiotropy (intercept=-0.005, SE=0.026, *p*=8.65e-01). The results of MR-PRESSO analysis also indicated that there was no directional pleiotropy and that there were no outliers for any IVs (*p*-global test=1.27e-01). In addition, we utilized the IVW radial method to identify potential outlying genetic variants and showed that there were no outliers (**Figure 4**). In the sensitivity analysis, the leave-one-out test showed that the causal estimation of genetically predicted LTL on MS risks was not influenced by a single SNP; no matter which SNP was removed, it would exert no fundamental effect on the results, implying the stability of our two-sample MR analysis (**Figure 5**).

## Discussion

In this study, the two-sample MR approach was used to evaluate the causal effect of LTL on the risk of MS, and we found that genetically determined short LTL was associated with an increased risk of MS in the European population. Our findings were valid and consistent in different MR methods and were stable in the sensitivity analysis. In addition, our results suggested that LTL contributed to the pathogenesis of MS. Considering the relationship between LTL and aging, our results also supported that biological age, not chronological age, was correlated with the risk of MS. In the future, attention should be shifted to LTL in the pathogenesis and treatment strategy of MS.

In order to investigate whether LTL has a similar relationship with other disorders of central nerves system, we further explored the causal relationship of LTL on neurodegenerative diseases, including amyotrophic lateral sclerosis (ALS), Parkinson’s disease (PD) and Alzheimer’s disease (AD). However, there was no significant association between genetic determined LTL and ALS risk (IVW method, *p*=0.784, 95% CI: 0.831-1.150), PD risk (IVW method, *p*=0.111, 95% CI: 0.960-1.481), and AD risk (IVW method, *p*=0.116, 95% CI: 0.674-1.044), respectively. These results suggest that LTL has a highly specific correlation with MS progression.

The natural process of aging, which is accompanied by increasing telomere attrition, is confronted with many problems, including the deteriorating immune system, the senescence of immune cells, and the higher risk of developing age-related neurological diseases mediated by autoimmunity and inflammation (28). T lymphocytes are representative immune cells in central nervous system dysfunctions and diseases (29). Studies have increasingly indicated that telomere shortening may play an essential role in the aging of T-cell immunity (30). Moreover, the number of studies concerning the role of senescent T lymphocytes in MS, which is characterized by neuroinflammation and neuronal disability triggered by immune cell infiltration, has also grown (4). Accelerated thymic deterioration and decreased release of naive T lymphocytes from the thymus have been observed in RRMS or primary progressive MS (PPMS) phenotypes (31, 32), highlighting the role of T-cell homeostasis, diminished response to novel antigens, and T lymphocyte senescence in the pathogenesis of MS (33).

Telomeres play a protective role in the processes of cell division, degradation and repair. They possess guanine-rich sequences at the end of chromosomes, which are highly sensitive to oxidative stress and inflammation (34). It has been described that TL can be influenced by genetic, environmental and behavioral factors (35). Telomere attrition related to aging can be promoted by increased oxidative stress and chronic inflammation. Previous observational studies have found an association between a higher content of DNA damage and protein oxidation markers and decreased TL (36).

Recently, the role of short telomeres in immune-mediated disorders, such as rheumatoid arthritis and systemic sclerosis, has been proposed (37–39). Currently, there is increasing awareness of the relationship between telomere and MS. It has been also reported that shorter LTL may increase the risk of developing MS in a case-control study (40). Additionally, shortened LTL is also related with clinical progression of MS (9). Telomere shortening is related to higher levels of chitinase 3-like protein 1, which is found in cerebrospinal fluid and serum at different disease stages of MS and is associated with a higher conversion rate from clinically isolated syndrome to relapsing-remitting MS (41, 42). A relationship has also been observed between elevated urinary 8-iso-PGF2α concentration, which serves as a lipid oxidation marker in demyelinating diseases (43, 44), and shorter lag-time of low-density lipoprotein oxidation, which indicates resistance to oxidation, and TL in MS patients (7). These findings suggest that shortened TL caused by enhanced oxidation is associated with the development of MS. In addition, when MS patients are administered antioxidants, such as vitamin E, the enhanced oxidation level can be rescued and the shortened TL maintained (45). Accelerated telomere length shortening during aging is presumably part of the altered immune response in MS and may contribute to neurodegeneration. In our MR analysis, LTL variants associated with traits that might influence the risk of MS independent of LTL were omitted to minimize the impact of confounders. All previous findings agree that shorter TL is related to the risk of developing MS, and our result is in accordance with previous studies.

The major strengths of our MR study include the valid IVs from the newest and largest GWAS database of LTL. In the current MR analysis, the GWAS data of LTL were obtained from a meta-analysis of 78,592 individuals from the European Network for Genetic and Genomic Epidemiology (ENGAGE) study and from the European Prospective Investigation into Cancer and Nutrition (EPIC) Cardiovascular Disease (CVD) and InterAct studies (13). The GWAS data of MS came from a genetic association study released by the International Multiple Sclerosis Genetics Consortium, which included 47,429 MS cases and 68,374 control individuals (20). Then, we established strict criteria to select IVs, and only LTL variants that were significantly related to the LTL measurements and that met the three core assumptions of MR analysis were chosen as IVs. Moreover, in the current study, to reduce bias in the causal estimation, different MR methods were adopted, and the results were valid and consistent in the sensitivity analysis (46). No clear heterogeneity, horizontal pleiotropy, or outliers were observed, suggesting the validity and robustness of our results. Our study determined that MS is a consequence of shortened LTL and a senescent immune system.

Despite the validity and stability of our MR results, there are several limitations of the current study. First, since the individuals included in the primary studies of LTL and MS GWAS were all European populations, our findings may not be extended to other populations, and our results should be stratified by various races. Second, only summary-level statistics were available in our MR analysis, and individual-level statistics were unreachable, limiting the stratified analysis of specific factors. Third, the subtype and severity of MS were not accessible, and the relationship between LTL and MS subtypes and severity could therefore not be estimated. Fourth, we have only excluded SNPs associated with already known confounders such as smoke, vitamin D level and BMI, some other unknown confounders that might influence the LTL-MS associations need to be further investigated. Finally, the LTL is determined by genetics as well as the environment, lifestyles and epigenetic modifications. It should be noted that our results could only partly explain the causal effect of LTL on MS.

## Conclusions

Our results suggest a potential causal effect of LTL on the risk of MS. Genetically predicted shorter LTL could increase the risk of MS in the European population. LTL should be noted and emphasized in the pathogenesis and treatment of MS. Large-scale studies in different populations are needed to elucidate the causal role of LTL in MS.

## Data availability statement

The raw data supporting the conclusions of this article will be made available by the authors, without undue reservation.

## Author contributions

KH and QL contributed to the conception and design of the study. QL and JH performed the statistical analysis. QL wrote the draft of the manuscript. All the authors polished the manuscript and critically revised the manuscript for valuable intellectual content. All the authors approved the final manuscript.

## Funding

This work was supported by the Science and Technology Innovation Program of Hunan Province, China (Grant No. 2021RC2023, KH), and the China Postdoctoral Science Foundation (Grant No. 2021M703638, KH).

## Conflict of interest

The authors declare that the research was conducted in the absence of any commercial or financial relationships that could be construed as a potential conflict of interest.

## Publisher’s note

All claims expressed in this article are solely those of the authors and do not necessarily represent those of their affiliated organizations, or those of the publisher, the editors and the reviewers. Any product that may be evaluated in this article, or claim that may be made by its manufacturer, is not guaranteed or endorsed by the publisher.

## References

[1] Compston A Coles A. Multiple sclerosis. Lancet (2002) 359:1221–31. doi: 10.1016/S0140-6736(02)08220-X 11955556

[2] Ostolaza Ibanez A Corroza Lavineta J Ayuso Blanco T. Immunosenescence: the role of age in multiple sclerosis. Neurologia (Engl Ed) (2022). doi: 10.1016/j.nrleng.2020.05.023 35260362

[3] Sanai SA Saini V Benedict RH Zivadinov R Teter BE Ramanathan M. Aging and multiple sclerosis. Mult Scler (2016) 22:717–25. doi: 10.1177/1352458516634871 26895718

[4] Fessler J Angiari S. The role of T cell senescence in neurological diseases and its regulation by cellular metabolism. Front Immunol (2021) 12:706434. doi: 10.3389/fimmu.2021.706434 34335619 PMC8317490

[5] Blackburn EH Epel ES Lin J. Human telomere biology: A contributory and interactive factor in aging, disease risks, and protection. Science (2015) 350:1193–8. doi: 10.1126/science.aab3389 26785477

[6] Blackburn EH. Switching and signaling at the telomere. Cell (2001) 106:661–73. doi: 10.1016/S0092-8674(01)00492-5 11572773

[7] Guan JZ Guan WP Maeda T Guoqing X Guangzhi W Makino N. Patients with multiple sclerosis show increased oxidative stress markers and somatic telomere length shortening. Mol Cell Biochem (2015) 400:183–7. doi: 10.1007/s11010-014-2274-1 25424527

[8] Miner AE Graves JS. What telomeres teach us about MS. Mult Scler Relat Disord (2021) 54:103084. doi: 10.1016/j.msard.2021.103084 34371369

[9] Hecker M Fitzner B Jager K Buhring J Schwartz M Hartmann A. Leukocyte telomere length in patients with multiple sclerosis and its association with clinical phenotypes. Mol Neurobiol (2021) 58:2886–96. doi: 10.1007/s12035-021-02315-y PMC8128833 33547621

[10] Krysko KM Henry RG Cree B Lin J University of California, S.F.M.S.E.T Caillier S. Telomere length is associated with disability progression in multiple sclerosis. Ann Neurol (2019) 86:671–82. doi: 10.1002/ana.25592 PMC7135931 31486104

[11] Buhring J Hecker M Fitzner B Zettl UK. Systematic review of studies on telomere length in patients with multiple sclerosis. Aging Dis (2021) 12:1272–86. doi: 10.14336/AD.2021.0106 PMC8279528 34341708

[12] Minelli C Del Greco MF van der Plaat DA Bowden J Sheehan NA Thompson J. The use of two-sample methods for mendelian randomization analyses on single large datasets. Int J Epidemiol (2021) 50:1651–9. doi: 10.1093/ije/dyab084 PMC8580269 33899104

[13] Li C Stoma S Lotta LA Warner S Albrecht E Allione A. Genome-wide association analysis in humans links nucleotide metabolism to leukocyte telomere length. Am J Hum Genet (2020) 106:389–404. doi: 10.1016/j.ajhg.2020.02.006 32109421 PMC7058826

[14] Interact, C Langenberg C Sharp S Forouhi NG Franks PW Schulze MB. Design and cohort description of the InterAct project: an examination of the interaction of genetic and lifestyle factors on the incidence of type 2 diabetes in the EPIC study. Diabetologia (2011) 54:2272–82. doi: 10.1007/s00125-011-2182-9 PMC4222062 21717116

[15] Langenberg C Sharp SJ Franks PW Scott RA Deloukas P Forouhi NG. Gene-lifestyle interaction and type 2 diabetes: the EPIC interact case-cohort study. PloS Med (2014) 11:e1001647. doi: 10.1371/journal.pmed.1001647 24845081 PMC4028183

[16] Danesh J Saracci R Berglund G Feskens E Overvad K Panico S. EPIC-heart: the cardiovascular component of a prospective study of nutritional, lifestyle and biological factors in 520,000 middle-aged participants from 10 European countries. Eur J Epidemiol (2007) 22:129–41. doi: 10.1007/s10654-006-9096-8 17295097

[17] Codd V Nelson CP Albrecht E Mangino M Deelen J Buxton JL. Identification of seven loci affecting mean telomere length and their association with disease. Nat Genet (2013) 45:422–427,427e421-422. doi: 10.1038/ng.2528 23535734 PMC4006270

[18] Elsworth B Lyon M Alexander T Liu Y Matthews P Hallett J. The MRC IEU OpenGWAS data infrastructure. bioRxiv (2020) 2020.2008.2010.244293. doi: 10.1101/2020.08.10.244293

[19] Hemani G Zheng J Elsworth B Wade KH Haberland V Baird D. The MR-base platform supports systematic causal inference across the human phenome. Elife (2018) 7. doi: 10.7554/eLife.34408 PMC5976434 29846171

[20] International Multiple Sclerosis Genetics, C. Multiple sclerosis genomic map implicates peripheral immune cells and microglia in susceptibility. Science (2019) 365. 10.1126/science.aav7188 PMC7241648 31604244

[21] Gombash SE Lee PW Sawdai E Lovett-Racke AE. Vitamin d as a risk factor for multiple sclerosis: Immunoregulatory or neuroprotective? Front Neurol (2022) 13:796933. doi: 10.3389/fneur.2022.796933 35651353 PMC9149265

[22] Vandebergh M Dubois B Goris A. Effects of vitamin d and body mass index on disease risk and relapse hazard in multiple sclerosis: A mendelian randomization study. Neurol Neuroimmunol Neuroinflamm (2022) 9. doi: 10.1212/NXI.0000000000001165 PMC8990978 35393342

[23] Manouchehrinia A Huang J Hillert J Alfredsson L Olsson T Kockum I. Smoking attributable risk in multiple sclerosis. Front Immunol (2022) 13:840158. doi: 10.3389/fimmu.2022.840158 35309300 PMC8927036

[24] Hone L Jacobs BM Marshall C Giovannoni G Noyce A Dobson R. Age-specific effects of childhood body mass index on multiple sclerosis risk. J Neurol (2022). doi: 10.1007/s00415-022-11161-4 PMC9363310 35532785

[25] Liu JJ Prescott J Giovannucci E Hankinson SE Rosner B Han J. Plasma vitamin d biomarkers and leukocyte telomere length. Am J Epidemiol (2013) 177:1411–7. doi: 10.1093/aje/kws435 PMC3676154 23660800

[26] Barragan R Ortega-Azorin C Sorli JV Asensio EM Coltell O St-Onge MP. Effect of physical activity, smoking, and sleep on telomere length: A systematic review of observational and intervention studies. J Clin Med (2021) 11. doi: 10.3390/jcm11010076 PMC8745211 35011817

[27] Muezzinler A Mons U Dieffenbach AK Butterbach K Saum KU Schick M. Body mass index and leukocyte telomere length dynamics among older adults: Results from the ESTHER cohort. Exp Gerontol (2016) 74:1–8. doi: 10.1016/j.exger.2015.11.019 26657493

[28] Kaszubowska L. Telomere shortening and ageing of the immune system. J Physiol Pharmacol (2008) 59 Suppl 9:169–86. 19261979

[29] Pilli D Zou A Tea F Dale RC Brilot F. Expanding role of T cells in human autoimmune diseases of the central nervous system. Front Immunol (2017) 8:652. doi: 10.3389/fimmu.2017.00652 28638382 PMC5461350

[30] Matthe DM Thoma OM Sperka T Neurath MF Waldner MJ. Telomerase deficiency reflects age-associated changes in CD4+ T cells. Immun Ageing (2022) 19:16. doi: 10.1186/s12979-022-00273-0 35321714 PMC8941756

[31] Hug A Korporal M Schroder I Haas J Glatz K Storch-Hagenlocher B. Thymic export function and T cell homeostasis in patients with relapsing remitting multiple sclerosis. J Immunol (2003) 171:432–7. doi: 10.4049/jimmunol.171.1.432 12817027

[32] Haegert DG. Multiple sclerosis: a disorder of altered T-cell homeostasis. Mult Scler Int (2011) 2011:461304. doi: 10.1155/2011/461304 22096637 PMC3197186

[33] Tomas-Ojer P Puthenparampil M Cruciani C Docampo MJ Martin R Sospedra M. Characterization of antigen-induced CD4+ T-cell senescence in multiple sclerosis. Front Neurol (2022) 13:790884. doi: 10.3389/fneur.2022.790884 35185762 PMC8852676

[34] Praveen G Sivaprasad M Reddy GB. Telomere length and vitamin B12. Vitam Horm (2022) 119:299–324. doi: 10.1016/bs.vh.2022.01.014 35337624

[35] Hecker M Buhring J Fitzner B Rommer PS Zettl UK. Genetic, environmental and lifestyle determinants of accelerated telomere attrition as contributors to risk and severity of multiple sclerosis. Biomolecules (2021) 11. doi: 10.3390/biom11101510 PMC8533505 34680143

[36] Chen JH Hales CN Ozanne SE. DNA Damage, cellular senescence and organismal ageing: causal or correlative? Nucleic Acids Res (2007) 35:7417–28. doi: 10.1093/nar/gkm681 PMC2190714 17913751

[37] Georgin-Lavialle S Aouba A Mouthon L Londono-Vallejo JA Lepelletier Y Gabet AS. The telomere/telomerase system in autoimmune and systemic immune-mediated diseases. Autoimmun Rev (2010) 9:646–51. doi: 10.1016/j.autrev.2010.04.004 20435169

[38] Heba AC Toupance S Arnone D Peyrin-Biroulet L Benetos A Ndiaye NC. Telomeres: New players in immune-mediated inflammatory diseases? J Autoimmun (2021) 123:102699. doi: 10.1016/j.jaut.2021.102699 34265700

[39] Liu S Chung MP Ley B French S Elicker BM Fiorentino DF. Peripheral blood leucocyte telomere length is associated with progression of interstitial lung disease in systemic sclerosis. Thorax (2021) 76:1186–92. doi: 10.1136/thoraxjnl-2020-215918 PMC9262637 34272332

[40] Habib R Ocklenburg S Hoffjan S Haghikia A Epplen JT Arning L. Association between shorter leukocyte telomeres and multiple sclerosis. J Neuroimmunol (2020) 341:577187. doi: 10.1016/j.jneuroim.2020.577187 32050150

[41] Hinsinger G Galeotti N Nabholz N Urbach S Rigau V Demattei C. Chitinase 3-like proteins as diagnostic and prognostic biomarkers of multiple sclerosis. Mult Scler (2015) 21:1251–61. doi: 10.1177/1352458514561906 25698171

[42] Perez-Miralles F Prefasi D Garcia-Merino A Gascon-Gimenez F Medrano N Castillo-Villalba J. CSF chitinase 3-like-1 association with disability of primary progressive MS. Neurol Neuroimmunol Neuroinflamm (2020) 7. doi: 10.1212/NXI.0000000000000815 PMC7357419 32611760

[43] Greco A Minghetti L Sette G Fieschi C Levi G. Cerebrospinal fluid isoprostane shows oxidative stress in patients with multiple sclerosis. Neurology (1999) 53:1876–9. doi: 10.1212/WNL.53.8.1876 10563647

[44] Greco A Minghetti L Levi G. Isoprostanes, novel markers of oxidative injury, help understanding the pathogenesis of neurodegenerative diseases. Neurochem Res (2000) 25:1357–64. doi: 10.1023/A:1007608615682 11059806

[45] Guan JZ Guan WP Maeda T. Vitamin e administration erases an enhanced oxidation in multiple sclerosis. Can J Physiol Pharmacol (2018) 96:1181–3. doi: 10.1139/cjpp-2018-0246 30092167

[46] Bowden J Del Greco MF Minelli C Davey Smith G Sheehan NA Thompson JR. Assessing the suitability of summary data for two-sample mendelian randomization analyses using MR-egger regression: the role of the I2 statistic. Int J Epidemiol (2016) 45:1961–74. doi: 10.1093/ije/dyw220 PMC5446088 27616674

